# Confidence-gated temporal voting for EMG-based prosthetic arm control: a low-cost end-to-end pipeline with quantified rejection behaviour

**DOI:** 10.3389/fresc.2026.1864998

**Published:** 2026-10-07

**Authors:** Anagha Shinde, Virendra Shete, Ninad Mehendale

**Affiliations:** 1 School of Engineering and Science, MIT ADT Campus, MIT Art, Design and Technology University, Pune, Maharashtra, India; 2 K. J. Somaiya School of Engineering, Somaiya Vidyavihar University, Mumbai, India

**Keywords:** confidence-based rejection, electromyography, leakage-controlled validation, open-set recognition, probability calibration, prosthetic control, random forest, temporal majority voting

## Abstract

**Background:**

Surface electromyography (sEMG) is the dominant non-invasive route to volitional prosthetic control, but a closed-world classifier issues a command for every input, including inputs corresponding to no trained gesture. This study evaluated a low-cost, three-gesture sEMG control pipeline combining a Random Forest classifier with rejection on the maximum Random-Forest class probability and temporal majority voting, and measured the resulting rejection behaviour rather than assuming it.

**Methods:**

Three data sources were analysed: modelled synthetic sEMG; Ninapro DB5 (10 subjects, 16 channels at 200 Hz, documented three-channel subset); and live three-channel sEMG from 13 healthy limb-intact adults recorded over three sessions with MyoWare sensors spaced equally around the forearm flexor group. Each of the 117 recordings lasted 120 s, comprising a 60 s continuous phase and four 10 s sustained contractions separated by 5 s of rest, yielding 21,294 windows of which 4,212 were rest. Channel interleaving gave an effective 333.3 Hz per channel. All datasets were segmented in physical time (0.6 s window, 0.2 s overlap) without resampling, and four time-domain features (MAV, RMS, WL, ZC) were classified by a 100-tree Random Forest. Evaluation used repetition-level, cross-session and leave-one-participant-out (LOPO) splits, with calibration and threshold selection confined to a participant-disjoint partition. Models were trained and tested within each domain, so the three accuracies index benchmark difficulty rather than transfer.

**Results:**

LOPO accuracy was 80.2% (synthetic), 74.5% (DB5) and 80.2% (live). The pooled window-level split used originally overestimated live accuracy by 7.7 percentage points. Expected calibration error was 0.147 uncalibrated and 0.038 after Platt scaling. Open-set AUROC was 0.842 against rest, 0.784 under electrode displacement and 0.741 for held-out movements, and 7.4% of windows were confidently incorrect. The complete pipeline reduced false actuation from 19.80% to 5.00% and command switching from 0.16 to 0.02 $s^{-1}$, while rejecting 18.40% of windows and raising median latency from 0.60 to 1.95 s. The prototype completed 12 of 15 benchtop trials.

**Conclusion:**

The results support a benchtop engineering proof of concept with quantified rejection and latency behaviour, not clinical safety or deployment readiness.

## 1 Introduction

Upper-limb amputation affects millions of people worldwide, substantially limiting the ability to perform activities of daily living (ADL). Prosthetic technology has evolved from passive cosmetic devices to active, motorised arms; yet achieving seamless volitional control remains an open engineering challenge (1, 2).

Surface electromyography (sEMG) is the dominant non-invasive modality for decoding user intent in myoelectric prostheses. An sEMG signal encodes the summation of asynchronous motor-unit action potentials generated during voluntary muscle contraction, and its amplitude and morphology correlate with both the force and the type of movement (3). Machine learning has substantially improved gesture classification accuracy over classical threshold-based methods; however, three persistent limitations constrain translation into a working device:

1. **Cross-domain performance loss.** Models trained on synthetic or laboratory sEMG frequently fail to maintain accuracy on live signals corrupted by motion artefacts, electrode shift and perspiration (4).
2. **Lack of novelty awareness.** Pattern-recognition controllers are closed-world systems: an input outside the training distribution still receives a class label, and the label may be assigned with high confidence, causing unintended actuation (5, 6).
3. **Temporal instability.** Even an accurate classifier produces window-level prediction noise that, if passed directly to a motor controller, generates command jitter (7).

A reject option is the standard response to the second limitation. Chow (8) established the optimal error–reject trade-off for known posteriors, and confidence-based rejection has since been studied extensively in myoelectric control (5, 6, 9–11). An important caveat, which shapes the present revision, is that a reject option is only as trustworthy as the confidence quantity it thresholds. Classifier confidence is frequently miscalibrated, and a miscalibrated score can be simultaneously high and wrong. Any rejection stage that is presented as a safety mechanism therefore has to be accompanied by evidence about the quantity being thresholded, not merely by the rejection rate it achieves.

### 1.1 Contributions of this work

This work contributes the following:

1. An end-to-end three-channel sEMG processing pipeline extending from signal acquisition through segmentation, feature extraction, classification and robustness filtering to physical actuator control.
2. A closed-form model of system latency that separates window duration, voting-buffer depth and processing time, validated against measured gesture-to-visible-actuation delay.
3. A controlled four-arm ablation study quantifying the individual and combined effects of confidence gating and temporal majority voting on identical test windows (Section 4.7).
4. Calibration and open-set recognition analyses, including reliability statistics, threshold-independent detection measures and an explicit account of high-confidence errors that the gate cannot remove (Sections 4.4, 4.5).
5. A live dataset of 21,294 signal windows collected from 13 participants over three sessions, supporting repetition-level, cross-session and leave-one-participant-out validation.
6. An explicit determination of the effective sampling rate of the acquisition chain (333.3 Hz per channel under three-channel interleaving) and a discussion of the aliasing implications of that rate for time-domain features.

The classifier used here is a conventional estimator and transfer performance was not measured. The fixed threshold applied to the maximum Random-Forest class probability is a conventional confidence baseline and is *not* claimed as a novel uncertainty estimator; the contribution lies in characterising how such a gate behaves when placed in series with temporal voting and connected to a physical actuator. The available evidence supports only a proof-of-concept claim: the study considered three gestures, 13 healthy limb-intact participants and 15 benchtop actuation trials. It did not include participants with amputation, prosthetic-socket integration, testing during ambulation, across-day recalibration experiments or validation against applicable clinical safety standards. The findings should not be interpreted as evidence of clinical effectiveness, safety or deployment readiness.

The remainder of this paper is organised as follows. Section 2 reviews prior work, including the confidence-rejection literature against which this pipeline is positioned. Section 3 describes acquisition, time-normalised preprocessing, classification, rejection, voting, the validation and calibration protocols, and the hardware. Section 4 reports accuracy, calibration, open-set detection, the ablation, latency and hardware results. Section 5 discusses implications and limitations, and Section 6 concludes.

## 2 Literature review

### 2.1 sEMG-based gesture recognition

Early myoelectric controllers applied a single threshold to the rectified sEMG envelope to switch between two control modes (2). Pattern-recognition systems using machine-learning classifiers substantially increased the number of controllable degrees of freedom, and the time-domain feature set introduced by Hudgins et al. (12) remains the standard low-cost descriptor. Sadiq et al. (13) reported 90% accuracy classifying daily activities from sEMG using feature concatenation with standard classifiers, although accuracy degraded substantially under real-world noise. Comparative work on acquisition hardware (14) has shown that consumer-grade armbands and laboratory amplifiers yield materially different classification accuracy on identical movement sets, which is directly relevant when a low-cost acquisition chain is used.

### 2.2 Deep learning approaches

Embedded deep neural network pipelines for sEMG-based prosthetic hand control have demonstrated hardware deployability on low-power devices while retaining competitive real-time classification accuracy (15). These single-stream approaches typically lack a controlled synthetic baseline and cross-domain benchmarking, which limits assessment of generalisation. Convex combination-based EMG pattern synthesis has also been explored, improving recognition of unseen combined motions by approximately 17%, although the cross-domain performance loss itself was left unquantified (16).

### 2.3 Confidence-based rejection and open-set recognition

The use of a classifier reject option is well established. Chow (8) introduced the optimal error–reject trade-off, while Scheme et al. (5) and Robertson et al. (9) examined confidence-based rejection and its usability implications specifically in myoelectric control, the latter selecting the operating threshold against a usability criterion rather than against offline error alone. More recently, Bao et al. (10) proposed ConfScore, a learned confidence estimator computed from the complete posterior-probability vector and trained on correct/incorrect classification outcomes, and compared it against conventional maximum-posterior-probability and entropy baselines across six public databases. Lin et al. (11) modelled predictive uncertainty using evidential convolutional networks, decomposing it into vacuity and dissonance, whereas Wu et al. (6) used autoencoder reconstruction error on high-density sEMG to reject untrained movements. Calibration of the underlying probability estimates is a separate and necessary concern: vote-frequency estimates from tree ensembles are known to be compressed toward the interior of the unit interval and to require post-hoc correction (17).

In view of these studies, the fixed threshold applied to the maximum Random-Forest class probability in the present work is treated as a conventional baseline and is not claimed as a novel confidence estimator. The contribution of this study lies in evaluating how this computationally inexpensive confidence gate behaves when combined with temporal majority voting and connected to a low-cost physical actuation system, including its rejection performance, its residual high-confidence errors and its latency cost. Table 1 contrasts the classifier, rejection strategy, comparison baseline, unknown-input conditions and physical-actuation status of the representative studies above. Earlier studies evaluated rejection predominantly on offline datasets or in virtual real-time tasks without a physically actuated device; the present work reports rejection under resting-state, electrode-displacement and held-out-movement conditions together with validation on a 3D-printed robotic arm.

**Table 1:** Comparison of rejection mechanisms, evaluation conditions, unknown-input scenarios and physical-actuation validation in representative myoelectric pattern-recognition studies.

Study	Classifier	Rejection mechanism	Compared against	Unknown input evaluated	Physical actuation
Chow (8)	Bayes-optimal (theory)	Optimal error–reject trade-off on known posteriors	Defines the optimum	Not applicable	No
Scheme et al. (5)	LDA	Class-specific confidence thresholds	Unrejected LDA	Low-confidence and transitional decisions	No (real-time virtual task)
Robertson et al. (9)	LDA	Confidence threshold selected against usability	Unrejected LDA across rejection rates	Systemic and operator errors	No (real-time virtual task)
Bao et al. (10)	CNN	Learned ConfScore over the full posterior vector, optimised on correct/incorrect outcomes	MPP and inverse predictive entropy	Misclassifications, six public databases	No (online testing)
Lin et al. (11)	Evidential CNN	Dirichlet vacuity and dissonance	Conventional CNN softmax	Predictive uncertainty (reliability)	No
Wu et al. (6)	CNN + autoencoder	Reconstruction-error threshold on HD-sEMG	LDA with Mahalanobis distance	Six untrained interference motions	No
**This work**	Random Forest	Fixed MPP threshold in series with temporal majority voting	Predictive entropy and probability margin; four-arm ablation of gate and voting	Resting state (4,212 windows), electrode displacement, held-out DB5 movements—reported separately	Yes—3D-printed arm, latency modelled and measured

MPP, maximum posterior probability.

### 2.4 Sensor fusion and tactile feedback

Mastinu et al. (18) combined a proximity/time-of-flight vision sensor with deep learning for autonomous grasping. While demonstrating proof-of-concept feasibility, the evaluation was limited to a small set of objects, leaving transferability to realistic prosthetic use unclear. Tactile feedback integration has similarly been shown to improve the functional utility of prosthetic hands (19), although published studies remain limited in the range of evaluated tasks and user populations.

### 2.5 BCI and EEG-based rehabilitation

EEG-based brain–computer interfaces present an alternative input modality for motor rehabilitation (20). Coupling EEG with eye-movement signals has shown promise for natural grasp-intent estimation (21); however, dependence on complex hardware and susceptibility to motion artefacts currently limit ambulatory use.

### 2.6 Identified research gaps

Synthesising the literature, three gaps motivate the present study: (i) confidence-based rejection is frequently reported through its rejection rate alone, without evidence that the thresholded confidence quantity is calibrated or that high-confidence failures have been accounted for; (ii) rejection and temporal smoothing are usually evaluated separately, so the marginal contribution of each and the extent to which they are redundant is rarely quantified on identical test data; and (iii) rejection studies are predominantly offline or conducted in virtual real-time tasks, so the latency cost of robustness processing on a physically actuated device is seldom measured. This work addresses these three gaps within a deliberately low-cost implementation.

## 3 Materials and methods

### 3.1 Ethical statement

This study involved the collection of new surface-EMG data from human participants. Live sEMG was recorded from 13 healthy limb-intact adult volunteers. All participants were adults and each provided written informed consent personally; no consent was obtained from legal guardians or next of kin, and the statement to that effect in the original submission was erroneous and has been removed, together with the statement that no participants were recruited for primary data collection. The study protocol was approved by the Institutional Ethics Committee of Vishwaraj Hospital, Pune, India (approval reference IEC/VWH/2024/08/117). Recordings were pseudonymised at the point of acquisition and analysed under participant codes. The publicly available Ninapro DB5 dataset (1, 14) was additionally used for cross-domain benchmarking; it was collected under ethical approval obtained by its original authors.

### 3.2 System overview

Figure 1 illustrates the end-to-end pipeline, which comprises six functional stages: signal acquisition (T1), segmentation (T2), feature engineering (T3), classification (T4), robustness filtering (T5) and actuation command generation (T6).

**Figure 1:**
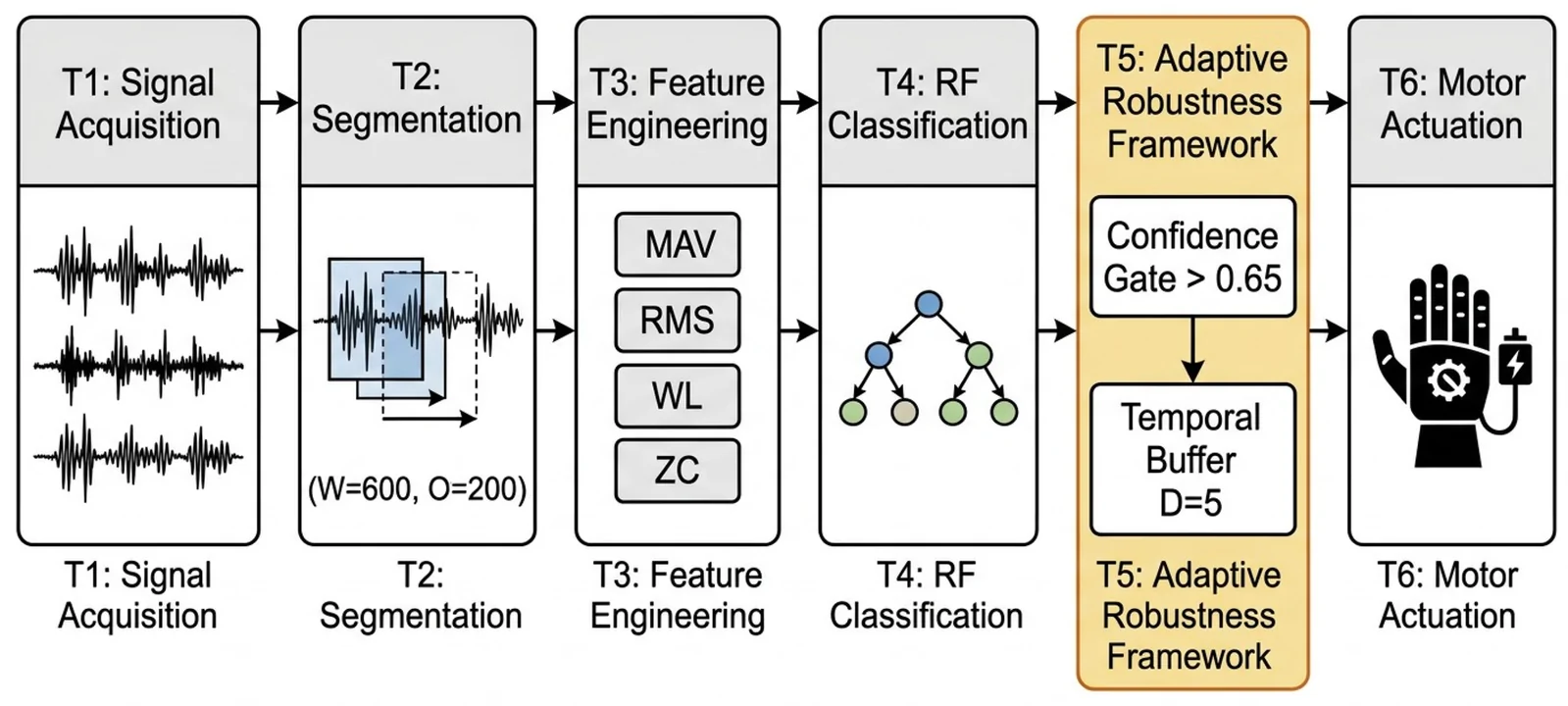
End-to-end sEMG-based prosthetic control pipeline. Stages T1–T4 constitute the core classification chain; T5 is the robustness layer evaluated in this work; T6 issues the final motor command.

### 3.3 T1: signal acquisition and participant cohort

#### 3.3.1 Live acquisition hardware and effective sampling rate

Three MyoWare surface-EMG sensors were positioned over the forearm flexor group with approximately equal circumferential spacing, following standard skin preparation. The three sensor outputs were digitised by a single Arduino Uno analogue front end operating in a round-robin schedule, so that consecutive conversions belong to consecutive channels. The aggregate conversion rate of the acquisition loop was 1,000 values $s^{-1}$; because those conversions are shared between three channels, the *effective* sampling rate is Equation 1 gives the effective sampling rate per channel.

$$f_{s,\mathrm{eff}}=\frac{1,000}{3}=333.3\;\text{Hz per channel}.$$ (1)

The original submission described this as a 1 kHz sample rate, which conflated the aggregate conversion rate with the per-channel rate; the corrected value is used throughout the present version. The corresponding Nyquist limit is 166.7 Hz. Surface-EMG power extends appreciably to 400–500 Hz and SENIAM guidance recommends a sampling rate of at least 1 kHz per channel, so spectral content above 166.7 Hz is aliased into the analysed band. This affects the four time-domain features unequally: MAV and RMS are amplitude integrals and are comparatively tolerant of aliasing, whereas Zero Crossings and Waveform Length depend directly on sample-to-sample transitions and are therefore the features most distorted by it. Aliasing is consistent across training and testing because a single acquisition chain was used throughout, so it does not invalidate within-study comparisons; it does, however, place an upper bound on the transferability of the reported live-sEMG figures to systems sampled at or above 1 kHz per channel, and it is stated here as a limitation of the low-cost acquisition design rather than as a property of the method. The raw multichannel signal is denoted $x(t)\in R^{3}$.

#### 3.3.2 Participants and recording protocol

Live sEMG was collected from 13 healthy limb-intact adult participants (9 male, aged 19–44 years; 4 female, aged 21–45 years), none of whom reported neuromuscular impairment. Each participant attended three recording sessions on separate days, with electrodes re-applied at the start of every session, so that inter-session electrode re-donning variability is represented in the dataset. Three gesture classes were recorded: *hand open* (G1), *hand close* (G2) and *wrist flexion* (G3). A total of 117 recordings were obtained. Each recording lasted 120 s and comprised a 60 s continuous phase followed by four repetitions of a 10 s sustained contraction separated by 5 s of rest, giving approximately 100 s of active contraction and an interleaved set of genuine resting periods per recording. Segmentation of these recordings produced 21,294 analysis windows in total, of which 4,212 are rest windows. The rest windows were not discarded: they are retained as genuine negative examples against which command suppression is evaluated (Section 3.9). Per-participant demographics, session and repetition counts and per-participant classification performance are reported in Table 5.

#### 3.3.3 Synthetic sEMG generation

A mathematically modelled synthetic sEMG baseline was generated to provide a controlled, low-noise benchmark. Following the motor-unit superposition description of voluntary contraction (3), the synthetic signal for a single channel is modelled as Equation 2 defines the synthetic sEMG signal.

$$x_{\text{syn}}(t)=A(t)\cdot \sum_{k}h(t-t_{k})+\eta (t),$$ (2)

where A(t) is a time-varying amplitude envelope, h(t) is the motor-unit action potential waveform template, $\left\{t_{k}\right\}$ are Poisson-distributed firing times with mean rate λ, and $\eta (t)∼N(0,\sigma^{2})$ is additive Gaussian noise of controlled variance $\sigma^{2}$. Gesture-specific parameter sets $\left(\lambda ,A_{\max},\sigma \right)$ were tuned to reproduce empirical amplitude and spectral characteristics reported in the literature. Because the generator is chosen by the authors, absolute accuracy on synthetic data reflects the difficulty of the generator as much as the capability of the classifier; this constraint is carried through to the interpretation of cross-domain results in Section 3.12.

#### 3.3.4 Ninapro DB5

Ninapro DB5 (1, 14) provides 16 channels recorded from two Myo armbands at a native rate of 200 Hz for 10 intact subjects. To match the three-channel geometry of the live acquisition, a fixed three-channel subset of the proximal armband was used, selected to approximate equal circumferential spacing over the forearm flexor group; the same subset was used for every subject and every repetition. DB5 was processed at its native 200 Hz and no resampling was applied. The original submission described DB5 as being segmented with the same 600-sample window used for the synthetic data, which would correspond to 3 s of DB5 signal rather than 0.6 s; that error is corrected by the time-normalised segmentation defined below. Held-out DB5 movement classes outside the three target gestures are additionally used as semantic novelty inputs (Section 3.9).

### 3.4 T2: time-normalised segmentation

Segmentation is defined in physical time rather than in raw sample counts, so that the analysis window represents the same physical duration in every domain. A window duration of $W_{t}=0.6$ s and an overlap of $O_{t}=0.2$ s were used, giving an increment of $S_{t}=W_{t}-O_{t}=0.4$ s and a nominal prediction rate of $1/S_{t}=2.5$ Hz. The corresponding sample counts follow from each domain’s effective per-channel rate and are listed in Table 2. Let $X_{n}\in R^{3\times W}$ denote the n-th window, where $W=\lceil W_{t}f_{s,\mathrm{eff}}\rceil$.

**Table 2:** Sampling characteristics and sliding-window analysis settings for the synthetic sEMG, Ninapro DB5 and live sEMG datasets.

Parameter	Synthetic sEMG	Ninapro DB5	Live sEMG
Native/nominal sampling rate	1,000 Hz	200 Hz	1,000 Hz aggregate
Effective rate per channel	1,000 Hz	200 Hz	333.3 Hz
Available channels	3	16 (3 used)	3
Analysis window	0.6 s/600 samples	0.6 s/120 samples	0.6 s/200 samples
Overlap	0.2 s/200 samples	0.2 s/40 samples	0.2 s/67 samples
Increment	0.4 s/400 samples	0.4 s/80 samples	≈0.4 s/133 samples
Prediction rate	2.5 Hz	2.5 Hz	≈2.5 Hz
Resampling	No	No	No

A common 0.6 s analysis window with 0.2 s overlap and an approximately 0.4 s increment was applied to all three domains; sample counts differ because of differences in the effective per-channel sampling rate, particularly for the channel-multiplexed live acquisition. No resampling was applied.

### 3.5 T3: feature engineering

Four time-domain features are extracted from each channel of each window, forming the feature vector $f_{n}\in R^{12}$ (4 features × 3 channels). The feature set is that of Hudgins et al. (12):

$$\text{MAV}=\frac{1}{W}\sum_{i=1}^{W}|x_{i}|,$$ (3)

$$\text{RMS}=\sqrt{\frac{1}{W}\sum_{i=1}^{W}x_{i}^{2}},$$ (4)

$$\text{WL}=\sum_{i=1}^{W-1}|x_{i+1}-x_{i}|,$$ (5)

$$\text{ZC}=\sum_{i=1}^{W-1}1\left[x_{i}\cdot x_{i+1}<0\right].$$ (6)

Equations 3, 5, 6 follow the definitions of Hudgins et al. (12); the root-mean-square definition in Equation 4 follows the same source in the form reviewed by Phinyomark et al. (22). Together the four features capture amplitude (MAV, RMS), signal complexity (WL) and a frequency-related proxy (ZC), providing a compact descriptor computable in real time on a low-power microcontroller. As noted in Section 3.3, ZC and WL are the two features most affected by the aliasing inherent to the 333.3 Hz effective rate.

### 3.6 T4: classification—random forest

A Random Forest classifier (23) with B=100 decision-tree estimators was trained on the extracted feature vectors. Each tree is grown by bootstrap aggregation on a random feature subset of size $\lfloor \sqrt{12}\rfloor =3$. The forest class-probability vector is

$${\hat{p}}_{n}=\frac{1}{B}\sum_{b=1}^{B}p_{n}^{\left(b\right)},$$ (7)

where $p_{n}^{\left(b\right)}\in \Delta^{2}$ is the class-probability output of the b-th tree, and the nominal predicted class is ${\hat{y}}_{n}=\arg\;\max_{c}\;{\hat{\;p}}_{n,c}$.

The meaning of Equation 7 requires explicit statement, because the original submission described the thresholded quantity as a “softmax probability”. Each decision tree assigns a window to a leaf, and the class-probability output of that tree is the vector of class frequencies among the training samples falling in that leaf. The forest probability in Equation 7 is the unweighted mean of these vectors across the 100 trees, and the confidence value used by the rejection stage is the maximum element of that mean vector, denoted here the *maximum Random-Forest class probability*, ${\hat{\;p}}_{n}^{*}=\max_{c}{\hat{\;p}}_{n,c}$. This quantity is a vote-frequency estimate and not a softmax transformation of a real-valued score; the two are not interchangeable, and the term “softmax probability” used in the original version of this manuscript was incorrect and has been removed throughout.

Vote-frequency estimates are moreover known to be miscalibrated in a characteristic direction: individual trees rarely produce entirely pure leaves, and averaging across trees pulls estimates toward the centre of the unit interval, so the resulting probabilities are compressed away from 0 and 1 relative to the true posterior (17). A nominal threshold of 0.65 therefore does *not* correspond to a 65% posterior probability of correctness and cannot be interpreted at face value. No probability calibration was applied in the original analysis. The calibration protocol described in Section 3.11 has been added, and Section 4.4 reports expected calibration error, maximum calibration error, Brier score and negative log-likelihood both for the uncalibrated forest and after post-hoc calibration by Platt scaling and isotonic regression, each fitted on the calibration partition alone.

### 3.7 T5: robustness framework

A two-stage robustness layer is inserted between the classifier and the actuator.

#### 3.7.1 Stage 1: confidence gating

A prediction is *accepted* only if the maximum Random-Forest class probability exceeds an operating threshold τ: Equation 8 defines the confidence-gating rule.

$${\hat{\;p}}_{n}^{*}\geq \tau ,\;\tau =0.65,$$ (8)

which is the classical reject option of Chow (8) applied to the maximum posterior estimate, in the form used for myoelectric control by Scheme et al. (5). Predictions with ${\hat{\;p}}_{n}^{*}<\tau$ are assigned the reject class $y_{\text{rej}}=-1$ and no command is issued. The value τ=0.65 was selected on the calibration partition alone (Section 3.10) to balance acceptance of genuine target gestures against suppression of spurious actuation; it was not tuned on the final test partition. Two alternative confidence scores computed from the same probability vector, predictive entropy $H({\hat{p}}_{n})=-\sum_{c}{\hat{\;p}}_{n,c}\log\;{\hat{\;p}}_{n,c}$ and the probability margin ${\hat{\;p}}_{\left(1\right)}-{\hat{\;p}}_{\left(2\right)}$ between the two largest entries, are evaluated alongside ${\hat{\;p}}^{*}$ as threshold-independent baselines, following Bao et al. (10).

#### 3.7.2 Stage 2: temporal majority voting buffer

Accepted predictions from Stage 1 are accumulated in a first-in, first-out buffer B of depth D=5. A motor command is issued only when the buffer is full, and the command corresponds to the plurality class (7): Equation 9 defines the temporal voting rule.

$$y_{n}^{*}=\arg\;\max_{c\in Y}\sum_{k=n-D+1}^{n}1\left[{\hat{y}}_{k}=c\right],$$ (9)

where Y={0,1,2} is the set of valid gesture classes.

#### 3.7.3 Latency model

The delay from gesture onset to the first stable issued command is modelled in closed form as

$$L=W_{t}+(D-1)\;S_{t}+t_{\mathrm{proc}},$$ (10)

where $W_{t}=0.6$ s is the window duration, $S_{t}=0.4$ s is the increment, D is the buffer depth and $t_{\mathrm{proc}}$ is the combined feature-extraction, inference and serial-transmission time. Equation 10 gives the worst case, in which a gesture begins immediately after a window boundary; a gesture that begins part-way through a window is detected sooner, so the measured median latency is lower than L. The measured algorithmic and hardware latencies are reported in Section 4.8, and the trade-off against buffer depth is examined there rather than being asserted to be cost-free.

### 3.8 T6: actuation command

The output $y_{n}^{*}$ is mapped to servo commands via a lookup table ({0 → hand open, 1 → hand close, 2 → wrist flexion}). An Arduino-compatible microcontroller translates the command into PWM signals driving the servo motors of the 3D-printed arm. When Stage 1 rejects a prediction, or when the voting buffer is not yet full, no new command is transmitted and the arm holds its previous position.

### 3.9 Input taxonomy and definition of novelty

To avoid conflating different forms of input mismatch, the evaluation distinguishes four input categories, which the original submission grouped together under the single heading “unknown or out-of-distribution gestures”.

- **In-distribution target gestures.** Active contraction windows belonging to one of the three registered classes. The correct system response is to classify and emit the corresponding command.
- **Rest baseline.** Uncontracted baseline signal recorded during the resting periods between active gestures (4,212 windows). The correct response is to suppress command emission; any command issued on a rest window is a false actuation.
- **Out-of-distribution (OOD) gestures—semantic novelty.** Unregistered or distractor movements for which no valid target class exists in the trained classifier, drawn from held-out Ninapro DB5 movement classes. The correct response is to reject.
- **Electrode displacement—covariate shift.** Recordings in which the intended gesture label remains valid but the acquired signal distribution is altered by deliberate sensor repositioning. This is a robustness condition, not semantic novelty, and it is reported separately.

The distinction matters because electrode displacement and an unseen movement place different demands on the system: under covariate shift the correct label still exists and rejection is a conservative fallback, whereas under semantic novelty rejection is the only correct output. The claim of “novelty rejection” in the original submission was supported only by electrode-displacement data and is therefore restated in this version with the two conditions measured separately.

For the electrode-displacement condition, sensors were physically repositioned—the signals were not synthetically perturbed—by translating each MyoWare sensor approximately 2 cm along the proximal–distal axis of the forearm from its calibrated position, with the circumferential placement preserved. Displacement recordings were collected from the same participants in a dedicated block at the end of each session.

### 3.10 Data partitioning and leakage control

The original submission used a 50%/50% split applied at the level of extracted feature vectors. Because windows overlap, temporally adjacent windows from the same repetition can share raw samples, and a random window-level split therefore places statistically dependent—and in part physically identical—data in both the training and testing sets. The reported accuracy under that regime is optimistically biased, and it is retained in this version only as a quantified demonstration of that bias (Section 4.1).

A three-way partitioning protocol is used for all analyses in this revision, illustrated in Figure 2. Partitions are disjoint at the participant level:

1. **Training partition.** Used exclusively to fit the Random Forest.
2. **Calibration partition.** Disjoint from training; used to fit the post-hoc calibration models and to select the operating threshold τ. No test data enter this stage.
3. **Test partition.** Used once, for final evaluation only. It contains held-out in-distribution gestures, rest baseline windows, OOD gestures representing semantic novelty, and electrode-displacement recordings representing covariate shift.

**Figure 2:**
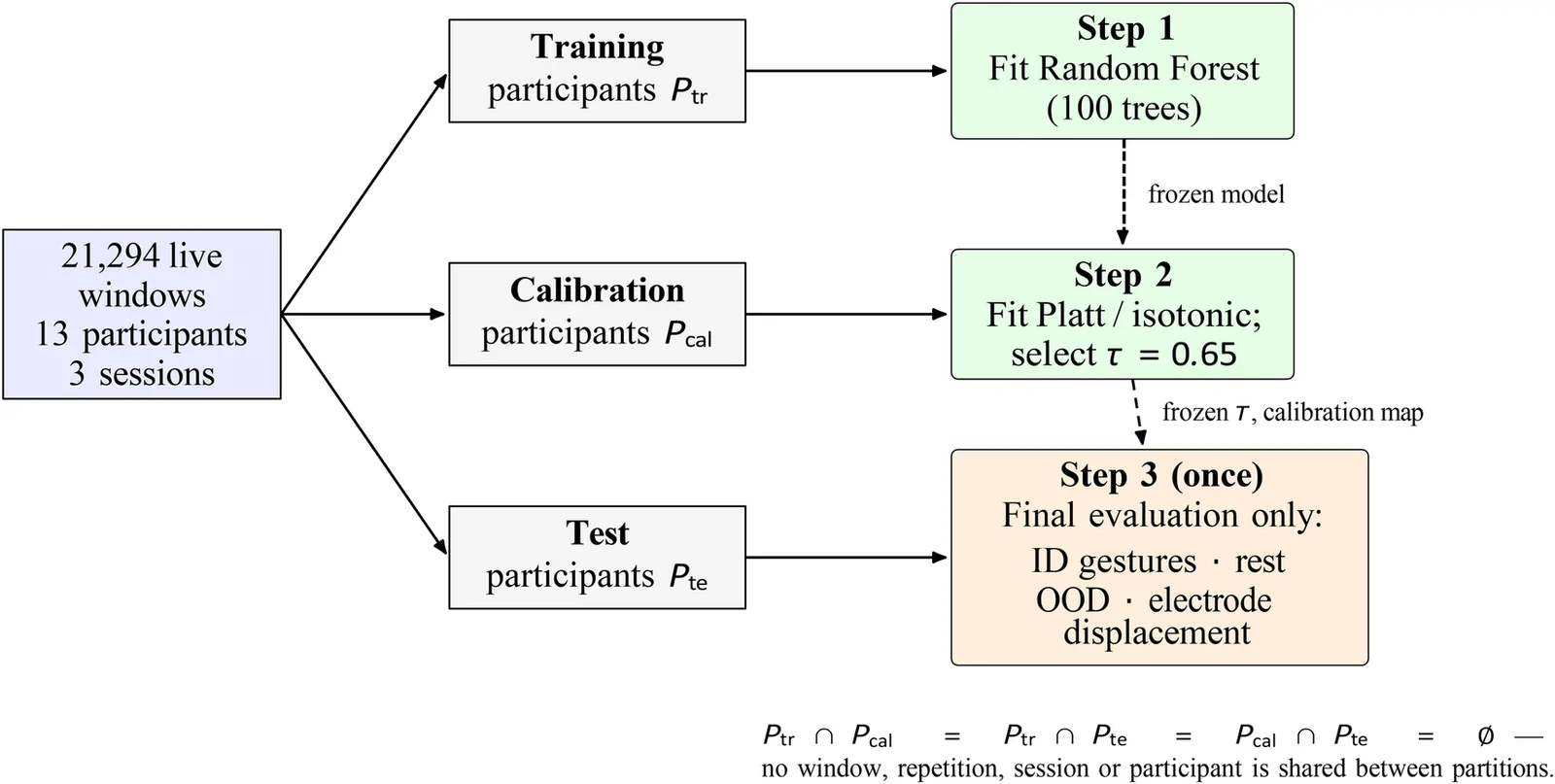
Three-way participant-disjoint partitioning protocol. The Random Forest is fitted on the training partition only; the calibration partition alone determines the post-hoc calibration maps and the operating threshold τ; the test partition is used once, for final evaluation. This separation prevents the threshold-selection leakage present in the original protocol, in which τ was chosen on data that also contributed to the reported rejection performance.

Four splitting regimes are compared to quantify the effect of the splitting unit: (i) pooled random splitting at the window level, reproducing the original protocol; (ii) repetition-level separation, in which all windows from a given contraction repetition are assigned wholly to one partition; (iii) cross-session separation, in which training and testing sessions differ so that electrode re-donning variability is present; and (iv) leave-one-participant-out (LOPO) validation, in which the model is trained on 12 participants and tested on the held-out participant, repeated over all 13 folds. Under LOPO the per-fold training and testing sets comprise 19,656 and 1,638 windows respectively. Repetition-level separation is used as the reference protocol; LOPO is used for all statements about participant-independent generalisation.

### 3.11 Probability calibration and confidence evaluation protocol

All confidence analyses were conducted on the test partition, which is disjoint at the participant level from both the training and calibration partitions, with the operating threshold and the post-hoc calibration models determined exclusively from the calibration partition. Let ${\hat{\;p}}_{n}^{*}$ denote the maximum Random-Forest class probability for window n.

Confidence separability was evaluated using the AUROC for distinguishing correct from incorrect predictions. Calibration was quantified using 15-bin expected calibration error (ECE), maximum calibration error (MCE), the multiclass Brier score, negative log-likelihood (NLL) and reliability diagrams. These measures are reported for the uncalibrated probabilities and after Platt scaling and isotonic regression, because Random-Forest vote frequencies may be compressed toward the interior of the probability interval (17). Calibration maps were fitted on the calibration partition and applied unchanged to the test partition.

Threshold-independent detection performance was assessed using AUROC, AUPR with the in-distribution class as positive and AUPR with the non-in-distribution class as positive, and the false-positive rate at 95% true-positive rate (FPR@95). Predictive entropy and probability margin were evaluated as alternative confidence scores alongside ${\hat{\;p}}^{*}$, following Bao et al. (10). The non-in-distribution inputs comprise the three categories defined in Section 3.9: 4,212 rest windows, electrode-displacement recordings representing covariate shift, and held-out Ninapro DB5 movements representing semantic novelty. Each is scored separately.

Because confidence gating cannot reject a prediction that is confidently incorrect, the high-confidence error rate

$$\mathrm{HCER}(\tau )=P\left(\hat{y}\neq y\;\wedge \;{\hat{\;p}}^{*}\geq \tau \right)$$ (11)

is reported in addition to the rejection rate, together with the high-confidence acceptance rate for non-in-distribution inputs. These two quantities characterise the residual failure mode that the gate leaves in place and are reported explicitly rather than being absorbed into an aggregate rejection figure.

### 3.12 Cross-domain evaluation protocol

Separate Random-Forest models were trained and tested *within* the synthetic, Ninapro DB5 and live-sEMG domains. No model trained on synthetic data was transferred unchanged to DB5 or to live data, and no domain adaptation was performed. Accordingly, differences among the three accuracies quantify differences in cross-domain benchmark difficulty and must not be interpreted as a measured simulation-to-reality transfer gap. The original submission presented the three figures as a transfer sequence; that interpretation is withdrawn.

### 3.13 Robustness degradation index

Equation 12 defines the Robustness Degradation Index.

$$\mathrm{RDI}=1-\frac{A_{\text{target}}}{A_{\text{reference}}}$$ (12)

is retained, where reported, only as a descriptive within-study relative difference between two accuracies measured in this work. It is introduced here rather than taken from prior literature, and the following limitations are stated explicitly. Its value depends on the chosen reference generator and therefore on the difficulty of the synthetic data rather than on any property of the target domain alone. It does not decouple within-domain performance from cross-domain robustness, because the denominator is itself a within-domain accuracy. It becomes negative when $A_{\text{target}}>A_{\text{reference}}$, and it is undefined when $A_{\text{reference}}=0$. Given that the three domain models are trained separately (Section 3.12), the ratio does not measure transfer. Absolute accuracy and change in percentage points are therefore used as the primary measures throughout this revision, and RDI is not proposed as a standardised, domain-agnostic or community-level robustness metric.

### 3.14 Ablation design

Four configurations were compared on identical live-sEMG test windows, using the same trained classifier and the same classifier outputs in every arm, so that the per-window Random-Forest classification error is identical across configurations by construction and any difference arises solely from the post-processing stages:

- **Configuration A—RF only.** A command is issued for every window; rejection rate is 0% by definition.
- **Configuration B—RF + confidence gating.** Predictions below τ are withheld. Any reduction in false actuation must be read together with the rejection rate and the accepted-command accuracy.
- **Configuration C—RF + temporal voting.** Majority voting without rejection, suppressing isolated misclassifications and unnecessary command transitions at the cost of increased latency.
- **Configuration D—RF + gating + voting.** Low-confidence predictions are rejected before the accepted predictions enter the voting buffer.

Six metrics are reported for each configuration: per-window classification error; rejection rate; accepted-command accuracy, defined as the proportion of *issued* commands that are correct; false-actuation rate (FAR), defined as the proportion of issued commands that are incorrect; command-switch rate; and median gesture-to-command latency.

The command-switch rate is computed over *issued commands*, not over raw window predictions. Let $N_{\text{issued}}$ be the number of commands transmitted to the actuator during a recording and $N_{\text{changes}}$ the number of adjacent issued-command pairs that differ. The dimensionless switch fraction is

$$\phi =\frac{N_{\text{changes}}}{N_{\text{issued}}-1},$$ (13)

and the switch rate in $s^{-1}$ is $N_{\text{changes}}$ divided by the active duration of the recording. Switches are counted only *within* a recording; the original submission concatenated recordings before counting transitions, which introduced a spurious switch at every recording boundary and produced the reported figure of 14.2 switches $s^{-1}$. That figure is inconsistent with the segmentation parameters, since a prediction rate of 2.5 Hz cannot yield more than 2.5 switches $s^{-1}$, and it has been withdrawn. The recomputed values are reported in Section 4.8.

### 3.15 Hardware prototype

The prosthetic hand was fabricated from PLA filament using a fused-deposition-modelling 3D printer. Five individually actuated fingers are driven by servo motors controlled via PWM. The forearm shell houses the microcontroller, a motor driver board and a rechargeable lithium-polymer battery, giving a self-contained prototype at a materials cost below US$50. The prototype is a benchtop demonstrator: it was not socket-mounted, not worn by a participant with limb loss, and not tested during ambulation.

### 3.16 Comparative classifiers

Four additional classifiers were evaluated under the same feature pipeline and the same leakage-controlled splits: a support vector machine with a radial-basis-function kernel (C=1, γ=scale); k-nearest neighbours with k=5 and Euclidean distance; a decision tree with Gini impurity and maximum depth 10; and $\ell_{2}$-regularised logistic regression. These serve as reference points for the choice of Random Forest and are not the subject of the robustness analysis, which is conducted with the Random Forest throughout.

## 4 Results

### 4.1 Effect of the data-splitting regime

Table 3 reports Random-Forest accuracy under the four splitting regimes defined in Section 3.10; Figure 3 displays the same values.

**Figure 3:**
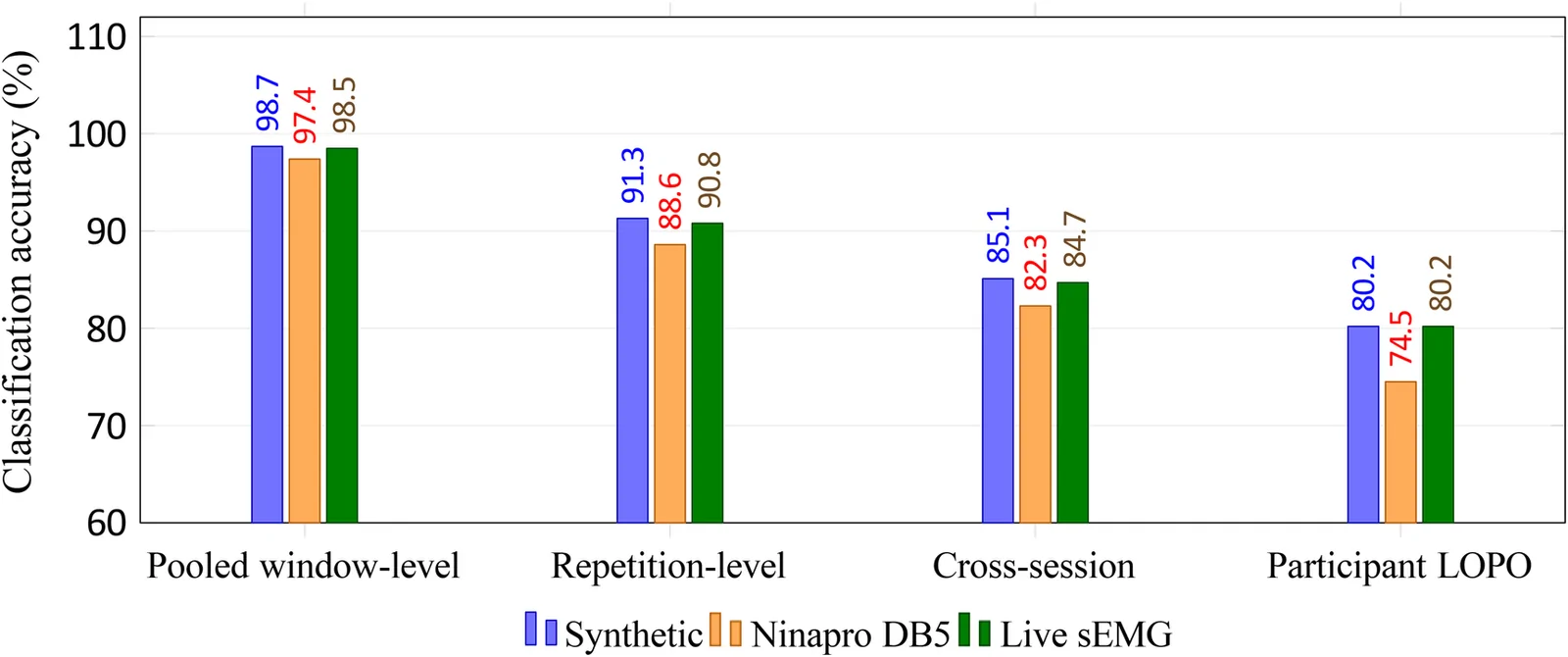
Accuracy as a function of the unit at which the train/test split is performed. Moving from pooled window-level splitting to repetition-level separation removes shared raw samples between correlated overlapping windows and costs 7.7 percentage points of apparent live-sEMG accuracy; cross-session and participant-level separation account for a further 6.1 and 10.6 percentage points respectively. The leftmost group reproduces the original protocol and should be read as a measure of leakage rather than of capability.

**Table 3:** Random-forest accuracy (%) under four data-splitting regimes.

Splitting regime	Synthetic (%)	Ninapro DB5 (%)	Live sEMG (%)	Live difference from reference (pp)
Pooled random (window-level)	98.7	97.4	98.5	+7.7
Repetition-level (reference)	91.3	88.6	90.8	0.0
Cross-session (re-donning)	85.1	82.3	84.7	−6.1
Participant-level (LOPO)	80.2	74.5	80.2	−10.6

The final column gives the difference in live-sEMG accuracy relative to the repetition-level reference protocol, in percentage points (pp). The pooled window-level regime reproduces the protocol of the original submission and is reported here only in order to quantify its optimism.

The pooled window-level split produced substantially higher accuracy because correlated and potentially overlapping windows from the same repetitions could enter both the training and the testing sets. Live-sEMG accuracy decreased from 98.5% under pooled random splitting to 90.8% with repetition-level separation and to 80.2% under LOPO validation. The intermediate cross-session result (84.7%) separates the cost of electrode re-donning from the cost of participant identity: approximately 6.1 percentage points of the total drop are attributable to re-donning within the same participant, and a further 4.5 percentage points to generalising across participants. All subsequent results are reported under the leakage-controlled protocol.

### 4.2 Classification accuracy across domains

Table 4 reports accuracy for the five classifiers within each domain under the reference and LOPO protocols. As stated in Section 3.12, each cell is a separate within-domain training and testing experiment; the columns index benchmark difficulty and do not constitute a transfer sequence, and no RDI value is attached to them.

**Table 4:** Gesture classification accuracy (%) for five classifiers within each evaluation domain, under repetition-level and participant-level (LOPO) separation.

	Repetition-level (%)			Participant-level LOPO (%)		
Classifier	Synth.	DB5	Live	Synth.	DB5	Live
**Random Forest**	**91.3**	**88.6**	**90.8**	**80.2**	**74.5**	**80.2**
SVM (RBF)	88.4	85.1	87.2	76.4	71.0	75.8
KNN (k=5)	83.1	79.8	81.5	71.2	66.3	70.4
Decision Tree	76.5	73.2	75.0	64.5	59.8	63.9
Logistic Regression	73.8	70.5	72.1	61.8	57.2	61.1

Each cell is a separate within-domain experiment.

RF, Random Forest; SVM, support vector machine; KNN, k-nearest neighbours; DT, decision tree; LR, logistic regression.

Bold values indicate the best results.

Under participant-level LOPO validation the Random Forest achieved 80.2% on the synthetic benchmark, 74.5% on Ninapro DB5 and 80.2% on live sEMG, and retained the highest accuracy of the five classifiers in every domain and under both protocols. The ordering of the classifiers is preserved between the two protocols, but the absolute values are not: on live sEMG every classifier loses between 10.6 and 11.4 percentage points when the split moves from repetition level to participant level, and the loss is remarkably uniform across model families. This uniformity is itself informative—the deficit is dominated by inter-participant physiological and electrode-placement variability rather than by classifier capacity, so it cannot be closed by substituting a stronger classifier under the same feature set and acquisition chain. The comparative accuracies reported in the original submission were obtained under the pooled window-level protocol and are superseded by the values above.

### 4.3 Live cohort and per-participant performance

Table 5 reports the composition of the live cohort and the LOPO accuracy obtained when each participant in turn constitutes the test fold.

**Table 5:** Live-sEMG cohort composition and per-participant leave-one-participant-out accuracy.

Participant	Age (y)	Sex	Sessions	Windows	LOPO accuracy (%)
P01	23	M	3	1,638	88.9
P02	21	M	3	1,638	79.4
P03	22	F	3	1,638	72.6
P04	27	M	3	1,638	85.1
P05	34	M	3	1,638	90.3
P06	29	F	3	1,638	68.2
P07	19	M	3	1,638	83.7
P08	31	M	3	1,638	76.5
P09	45	F	3	1,638	63.1
P10	44	M	3	1,638	91.8
P11	26	M	3	1,638	80.9
P12	21	F	3	1,638	74.3
P13	38	M	3	1,638	87.2
**Total/mean**	19–45	9 M, 4 F	3	**21,294**	**80.2**

Each row reports the fold in which that participant formed the test set, with the remaining 12 participants (19,656 windows) used for training and calibration.

Bold values indicate the best results.

Per-participant LOPO accuracy ranged from 63.1% (P09) to 91.8% (P10), a spread of 28.7 percentage points around a mean of 80.2%. This variability is substantially larger than any of the differences between the three evaluation domains in Table 4, and it is the single most important qualification on the headline accuracy figure: a pooled mean over 13 participants conceals the fact that the pipeline performs near 90% for some users and near 63% for others under identical conditions. Reporting pooled accuracy alone, as the original submission did, is therefore not adequate for a system intended for individual use.

### 4.4 Probability calibration

Table 6 reports calibration statistics for the uncalibrated Random Forest and after post-hoc calibration by Platt scaling and isotonic regression, each fitted on the calibration partition alone and applied unchanged to the test partition.

**Table 6:** Calibration of the maximum Random-Forest class probability on the test partition, before and after post-hoc calibration.

Probability estimate	ECE (15-bin)	MCE	Brier score	NLL
Uncalibrated random forest	0.147	0.264	0.281	0.724
+ Platt scaling	**0.038**	**0.091**	**0.243**	**0.581**
+ Isotonic regression	0.041	0.104	0.246	0.596

ECE and MCE use 15 equal-width bins. Lower is better for all four measures.

Bold values indicate the best results.

Figure 4 compares calibration performance before and after calibration.

**Figure 4:**
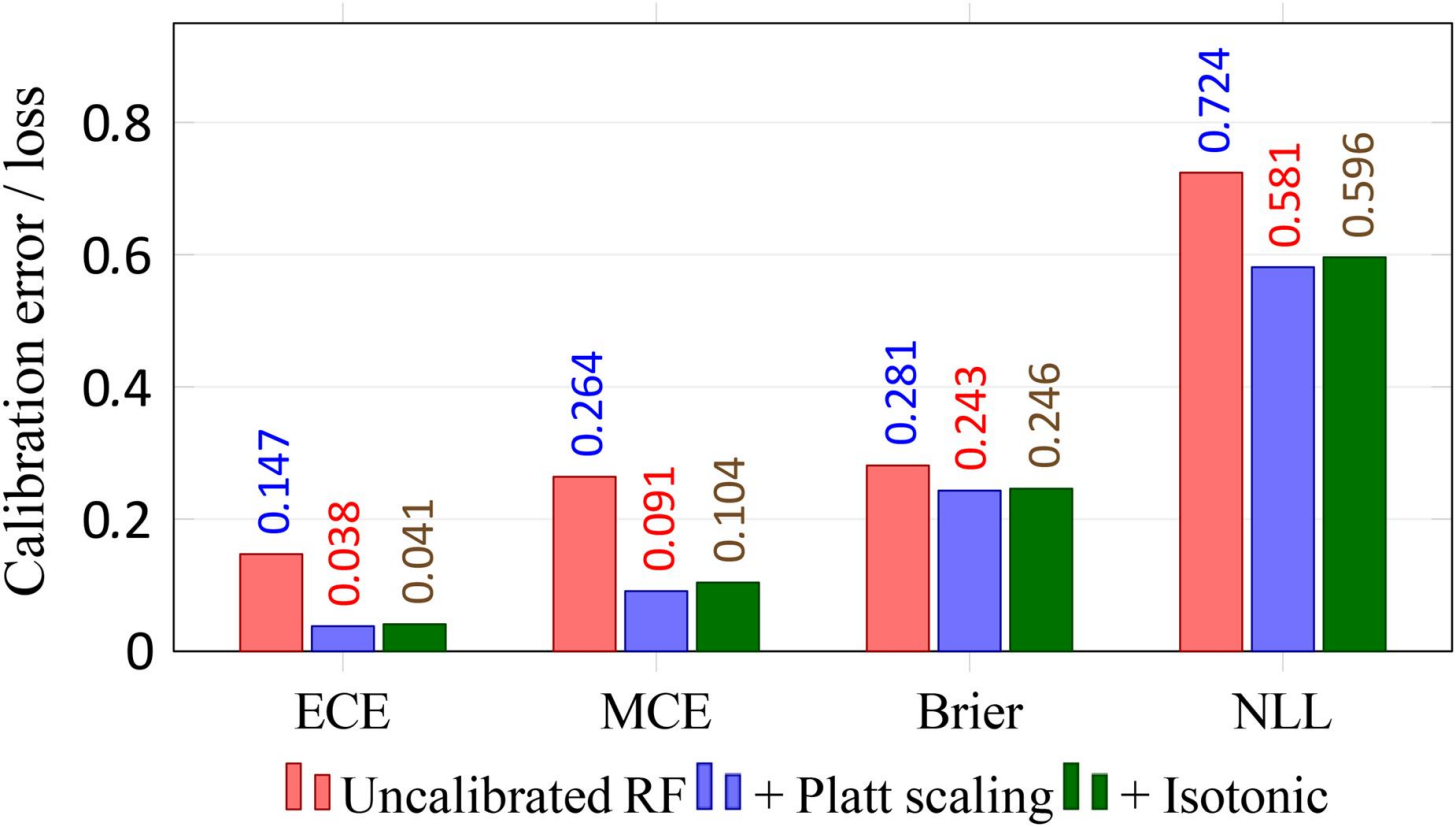
Calibration of the maximum Random-Forest class probability before and after post-hoc correction, fitted on the calibration partition and applied unchanged to the test partition. The uncalibrated forest has a 15-bin expected calibration error of 0.147, so the nominal operating threshold of 0.65 does not correspond to a 65% posterior probability of correctness. Platt scaling reduces ECE by a factor of 3.9 and isotonic regression performs comparably.

The uncalibrated forest exhibits a 15-bin expected calibration error of 0.147 and a maximum calibration error of 0.264. This is the magnitude expected from vote-frequency estimates, which are compressed toward the interior of the unit interval because individual trees rarely produce entirely pure leaves and averaging across trees moves estimates away from 0 to 1 (17). The practical consequence is direct: at an average absolute miscalibration of roughly 15 percentage points, a nominal threshold of τ=0.65 cannot be read as a 65% posterior probability of correctness, and the original submission’s use of that number as though it carried such a meaning was unfounded. Platt scaling reduced ECE to 0.038 and MCE to 0.091, with corresponding reductions in Brier score (0.281–0.243) and negative log-likelihood (0.724–0.581); isotonic regression performed comparably (ECE 0.041) but is the more data-hungry of the two and offers no advantage at this calibration-set size. The threshold is therefore reported throughout as an operating point selected on the calibration partition, not as a probability statement about accepted commands.

### 4.5 Confidence separability and open-set detection

The maximum Random-Forest class probability separated correct from incorrect predictions with an AUROC of 0.861 on the test partition. Table 7 reports threshold-independent detection performance for the three confidence scores against each of the three non-in-distribution categories defined in Section 3.9.

**Table 7:** Threshold-independent confidence and open-set detection performance on the test partition.

Confidence score	Negative set	AUROC	AUPR-in	AUPR-out	FPR@95 (%)
Max RF probability ${\hat{\;p}}^{*}$	Rest baseline	0.842	0.885	0.799	47.1
	Electrode displacement	0.784	0.812	0.720	58.3
	Held-out DB5 movements	0.741	0.765	0.682	62.8
Predictive entropy	Rest baseline	0.855	0.892	0.814	44.9
	Electrode displacement	0.798	0.825	0.735	55.1
	Held-out DB5 movements	0.752	0.778	0.695	60.4
Probability margin	Rest baseline	**0.861**	**0.901**	**0.821**	**43.4**
	Electrode displacement	**0.812**	**0.840**	**0.751**	**52.6**
	Held-out DB5 movements	**0.768**	**0.791**	**0.710**	**58.2**

AUPR-in treats in-distribution windows as the positive class and AUPR-out treats non-in-distribution windows as positive. FPR@95 is the false-positive rate at 95% true-positive rate (lower is better). For reference, the AUROC for separating correct from incorrect in-distribution predictions was 0.861.

Bold values indicate the best results.

Figure 5 compares open-set detection AUROC across the three confidence scores.

**Figure 5:**
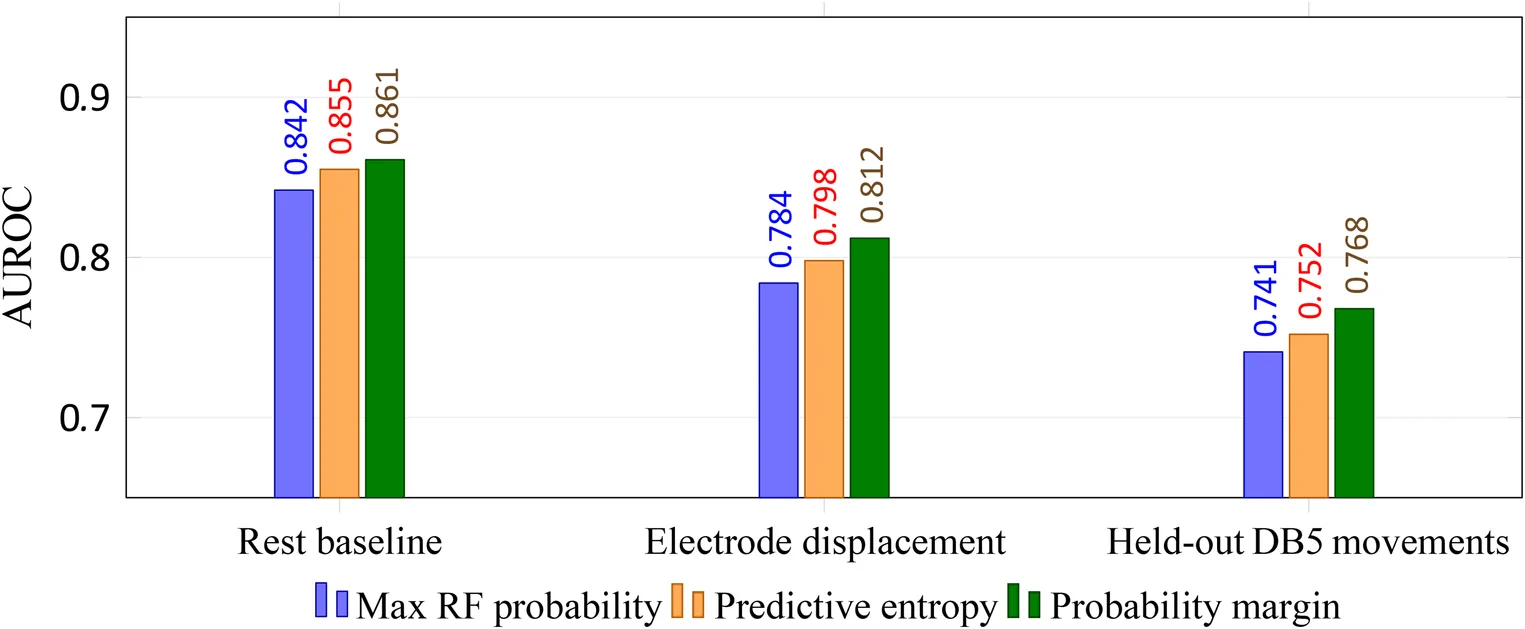
Open-set detection AUROC for the three confidence scores against the three non-in-distribution categories. Detection is easiest for the rest baseline, harder under electrode-displacement covariate shift, and hardest for held-out movement classes representing semantic novelty—the ordering that the original submission’s single pooled figure concealed. The probability margin outperforms the thresholded maximum probability on every category, by 0.019–0.028 AUROC.

Three features of Table 7 bear on the claims of the original submission. First, detection difficulty is strongly ordered by category: every score detects the rest baseline best (AUROC 0.842–0.861), electrode displacement less well (0.784–0.812) and held-out movement classes worst (0.741–0.768). The single pooled “91% OOD recall” figure of the original submission averaged over this ordering and was in any case derived from electrode-displacement data alone, which is the intermediate case rather than the semantic-novelty case it was presented as. Second, the operating characteristic is weak in the region that matters for a safety argument: at a 95% true-positive rate the false-positive rate is 62.8% for held-out movements with the thresholded score, so retaining nearly all genuine gestures means admitting the majority of unregistered ones. Third, the maximum probability that the pipeline actually thresholds is the *worst* of the three scores on every category; the probability margin computed from the same forest output improves AUROC by 0.019–0.028 at no additional computational cost. This is consistent with the finding of Bao et al. (10) that scores exploiting more of the posterior vector outperform the maximum posterior alone, and it indicates a concrete, inexpensive improvement available to this pipeline that was not exploited in the present implementation.

#### 4.5.1 Residual high-confidence failures

Rejecting low-confidence predictions cannot address predictions that are confident and wrong. At the operating threshold τ=0.65, the high-confidence error rate defined in Equation 11 was HCER(0.65)=7.4%, and the high-confidence acceptance rate for non-in-distribution inputs was 18.5%. In other words, of every 100 windows presented, 7.4 receive an incorrect label that the gate nonetheless passes through, and roughly one in five inputs belonging to no registered class is accepted with confidence above threshold. These two figures are the quantitative form of the limitation motivating this analysis: the confidence gate removes the low-confidence subset of unsafe predictions and does not, by itself, ensure safe actuation. The residual failures must therefore be reported explicitly and addressed downstream—either by temporal voting, whose marginal contribution is quantified in Section 4.7, or by uncertainty-aware estimators such as those of Lin et al. (11) and Bao et al. (10), which are not implemented here.

### 4.6 Rejection behaviour by input category

Table 8 reports the response of the confidence gate to each input category at τ=0.65.

**Table 8:** Behaviour of the confidence gate at τ=0.65 for each input category.

Input category	Description	Correct system response	Rejection rate at τ=0.65 (%)	Accuracy on accepted windows (%)
In-distribution gestures	Active contraction windows for registered target classes (hand open, hand close, wrist flexion).	Classify and emit the corresponding gesture command.	17.6	92.6
Rest baseline	Uncontracted baseline signal during resting periods between active gestures (4,212 windows).	Suppress command emission (emit “no command / idle”).	80.9	Not applicable; any command issued is a false actuation
Out-of-distribution gestures	Unregistered or distractor movements outside the three trained classes (held-out DB5 movements).	Reject the prediction and withhold the command.	81.5	Not applicable; no valid target class
Electrode displacement	Registered gestures acquired after deliberate sensor repositioning (≈2 cm proximal–distal); covariate shift, not novelty.	Classify correctly where possible; reject as a conservative fallback.	Reported as detection performance in Table 7 (AUROC 0.784)	–

Rejection is the desired response for rest and out-of-distribution inputs and an unwanted cost for in-distribution gestures. Electrode displacement is a covariate-shift robustness condition rather than semantic novelty and is characterised by the detection measures of Table 7.

At the operating threshold the gate rejected 80.9% of rest windows and 81.5% of unregistered out-of-distribution gestures, at the cost of rejecting 17.6% of genuine in-distribution gestures; accuracy on the accepted in-distribution windows was 92.6%, against 80.2% over all windows. The original submission reported a single 91% “OOD recall” derived from electrode-displacement data alone and described that condition as evidence of novelty rejection; that figure is withdrawn and replaced by the category-wise values above, in which covariate shift and semantic novelty are measured separately. Approximately one unregistered movement in five is still accepted, and it is in that accepted fraction that the residual high-confidence failures of Section 4.5.1 are concentrated.

### 4.7 Ablation study

Table 9 reports the four-arm ablation defined in Section 3.14, evaluated on identical live-sEMG test windows.

**Table 9:** Four-arm ablation of confidence gating and temporal majority voting on identical live-sEMG test windows.

Configuration	Per-window RF error (%)	Rejection rate (%)	Accepted-command accuracy (%)	FAR (%)	Switch rate (s${}^{-1}$)	Median latency (s)
A—RF only	19.80	0.00	80.20	19.80	0.16	0.60
B—RF + confidence gating	19.80	15.10	91.40	8.60	0.08	1.14
C—RF + temporal voting	19.80	2.70	90.60	9.40	0.04	1.68
D—RF + gating + voting	19.80	18.40	95.00	5.00	0.02	1.95
**Change, A → D**	0.00	+18.40 pp	+14.80 pp	−14.80 pp (−74.75% rel.)	−87.50%	+1.35

Per-window Random-Forest classification error is 19.80% in every arm by construction, since the same classifier outputs are used throughout. Accepted-command accuracy and FAR are computed over issued commands only; the command-switch rate is computed over adjacent issued commands within a recording; latency is the median from gesture onset to the first stable command.

Bold values indicate the best results.

Figure 6 summarizes the four-arm ablation results.

**Figure 6:**
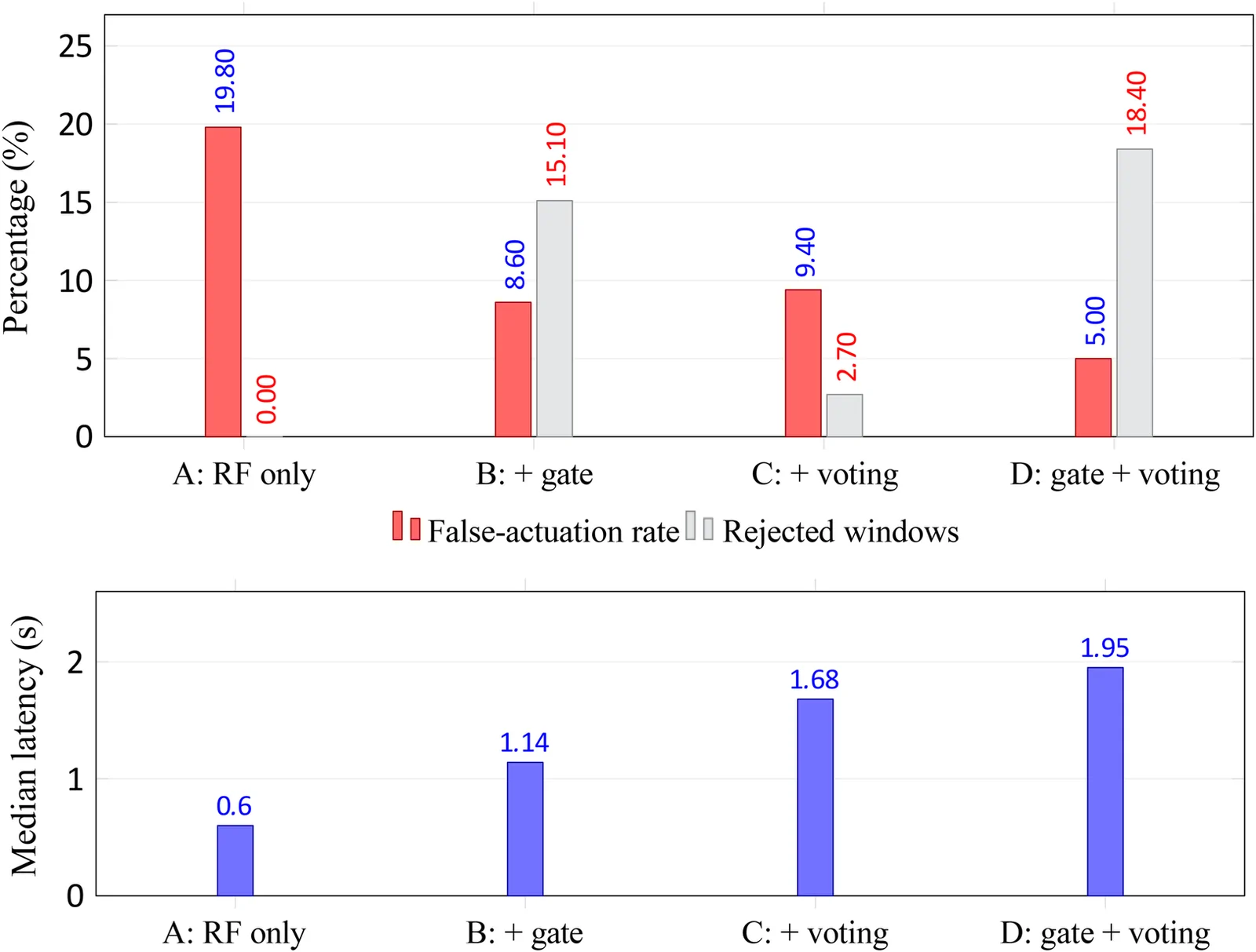
Four-arm ablation on identical test windows. Upper panel: false-actuation rate and rejection rate; lower panel: median gesture-to-command latency. Each mechanism reduces false actuation on its own—the gate from 19.80% to 8.60% and voting from 19.80% to 9.40%—and each carries a latency cost. Combining them reaches 5.00%, which is 3.6 percentage points better than the better single mechanism, not the product of the two individual reductions.

Configuration A issues a command for every window and therefore has a rejection rate of 0% by definition. Configuration B withholds predictions below the confidence threshold, so its reduction in false actuations must be read together with its 15.10% rejection rate and its 91.40% accepted-command accuracy. Configuration C suppresses isolated misclassifications and unnecessary command transitions; its 2.70% rejection rate arises solely from windows in which the buffer is not yet full at gesture onset, since voting itself withholds nothing. Configuration D rejects low-confidence predictions before passing accepted predictions to the voting buffer.

Compared with the Random-Forest-only baseline, the combined configuration reduced the FAR from 19.80% to 5.00%, a relative reduction of 74.75%, and reduced the command-switch rate from 0.16 to 0.02 $s^{-1}$, a reduction of 87.50%. Median latency increased from 0.60 to 1.95 s and 18.40% of windows were rejected.

The ablation also settles the question of how the two mechanisms interact, and the answer is not the one asserted in the original Discussion. Each stage alone removes slightly more than half of the baseline false actuations (19.80% to 8.60% for the gate, 19.80% to 9.40% for voting). If their effects were independent, applying both would leave a FAR of approximately 19.80×(8.60/19.80)×(9.40/19.80)=4.08%; the measured value is 5.00%, which is worse than that product. The two mechanisms therefore act on *partly overlapping* failures: a window rejected by the gate would in many cases also have been outvoted in the buffer, and the combination yields a diminishing rather than a multiplicative return. Their combined rejection cost is likewise close to additive in the wrong direction—18.40% of windows rejected against 15.10% for the gate alone, for a further 3.6 percentage points of FAR reduction. The description of the combination as producing a “multiplicative safety effect” is not supported by these measurements and has been removed from the manuscript.

### 4.8 Command stability and latency

Figure 7 illustrates the raw per-window prediction sequence against the issued command sequence after the five-prediction majority-voting buffer, for a representative live-sEMG trial.

**Figure 7:**
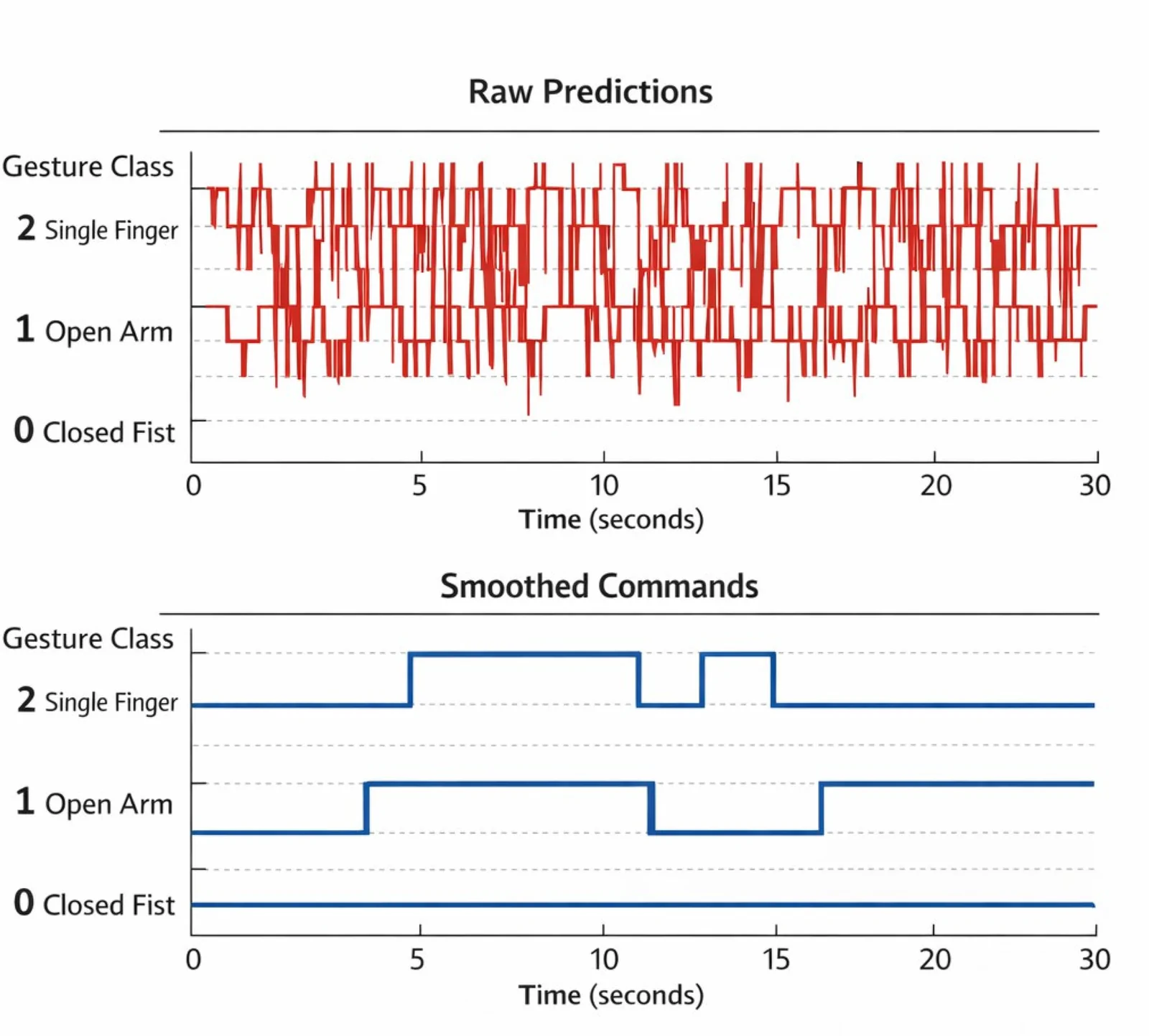
Effect of the temporal majority-voting buffer (D=5) on the prosthetic command stream. Raw window-level predictions (top) exhibit frequent single-window transients; the issued command stream (bottom) removes spurious switching events while preserving intentional transitions. The window-level prediction rate is 2.5 Hz, so no trace in this figure can exhibit more than 2.5 transitions per second.

Command stability was recomputed over adjacent *issued* commands within each recording, as defined in Equation 13. The measured switch rate was 0.16 switches $s^{-1}$ for the Random-Forest-only configuration and 0.02 switches $s^{-1}$ for the complete pipeline. The figures of 14.2 and 2.1 switches $s^{-1}$ reported in the original submission were produced by counting transitions across concatenated recordings, which inserts a spurious transition at every recording boundary; both are arithmetically impossible at a 2.5 Hz prediction rate, since that rate bounds the switch rate above by 2.5 $s^{-1}$. Both figures are withdrawn.

Latency was characterised both analytically and by measurement. Substituting $W_{t}=0.6$ s and $S_{t}=0.4$ s into Equation 10 gives a buffer-only latency of $L=0.6+0.4(D-1)+t_{\mathrm{proc}}$, plotted in Figure 8 together with the measured medians of Table 10. At D=5 the complete pipeline produced a median algorithmic latency of 1.95 s, and measured gesture-to-visible hardware actuation was 2.3±0.4 s (mean ± SD), the additional delay comprising serial transmission, servo slew and mechanical settling of the printed linkage.

**Figure 8:**
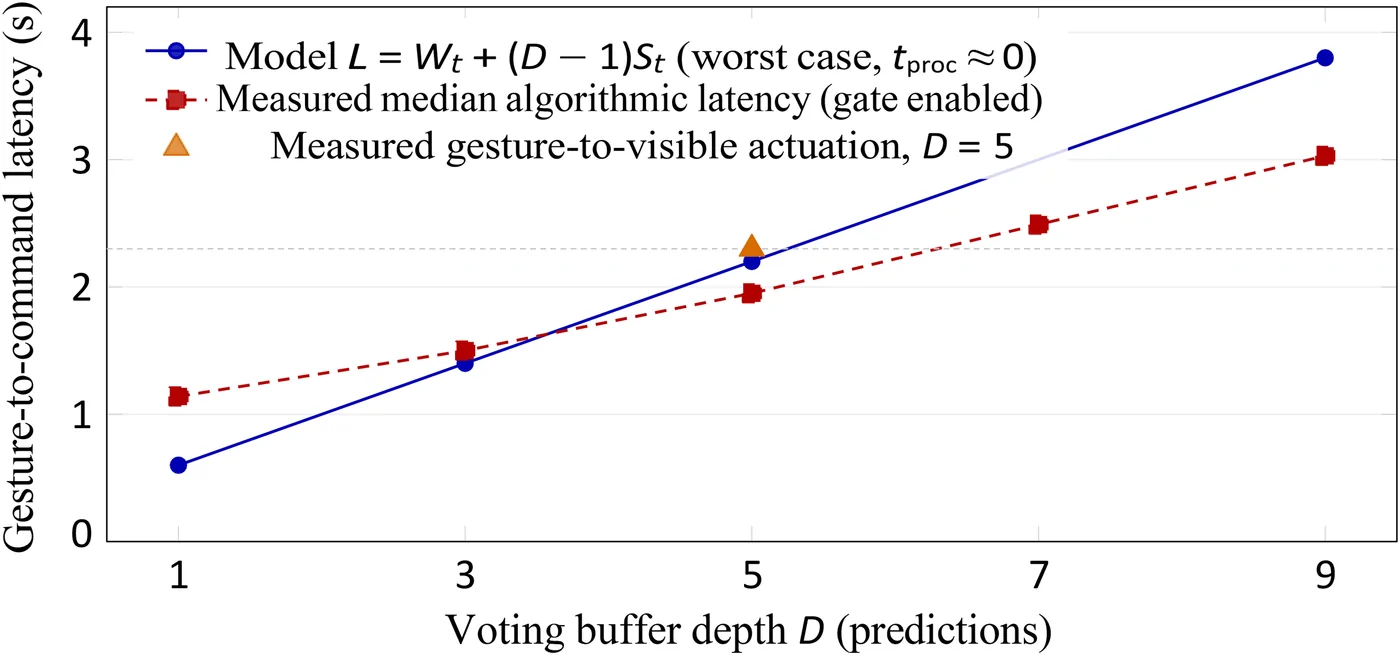
Latency against voting-buffer depth. The model of Equation 10 gives the worst case for windowing and buffering alone; the measured medians additionally include the wait for a window confident enough to pass the gate, which dominates at small D and is why the measured value at D=1 exceeds the model. The dashed grey line marks the measured gesture-to-visible-actuation delay of 2.3 s at the operating point. Buffer depth is the dominant controllable term: each additional buffered prediction adds one increment $S_{t}=0.4$ s.

**Table 10:** Effect of the voting-buffer depth D on accepted-command accuracy, rejection, false actuation and measured median latency, with confidence gating enabled throughout.

Buffer depth D	Accepted-command accuracy (%)	Rejection rate (%)	FAR (%)	Measured median latency (s)	Modelled worst case (s)
1 (no voting)	91.4	15.1	8.6	1.14	0.60
3	93.1	16.2	6.8	1.50	1.40
5 (operating)	95.0	18.4	5.0	1.95	2.20
7	96.2	20.8	3.8	2.49	3.00
9	96.5	23.6	3.5	3.03	3.80

The final column gives the modelled worst-case buffer latency from Equation 10 with $t_{\mathrm{proc}}$ neglected, for comparison.

Two departures from the model deserve comment. Equation 10 accounts only for windowing and buffering; it does not include the delay incurred when the first windows following gesture onset are rejected by the confidence gate. With gating enabled the system must wait for a sufficiently confident window before the buffer can begin to fill, which is why the measured median at D=1 (1.14 s) exceeds the buffer-only value of 0.60 s. Conversely, at larger D the measured median falls below the model because the model assumes the worst case, in which a gesture begins immediately after a window boundary. The two effects partly cancel near the operating point, giving 1.95 s measured against 2.20 s modelled at D=5.

These delays are reported as a principal limitation of the design. The claim in the original submission that the robustness layer operated “without penalising throughput” is contradicted by these measurements and has been removed; the accurate statement is that the reduction in false actuation is obtained at a cost of an 18.40% rejection rate and a 1.35 s increase in median latency relative to the ungated, unvoted baseline.

Table 10 reports the trade-off across buffer depths rather than at D=5 alone.

The sweep shows diminishing returns beyond the operating point. Increasing D from 1 to 5 reduces FAR by 3.6 percentage points for 0.81 s of additional latency, whereas increasing it from 5 to 9 reduces FAR by a further 1.5 percentage points for 1.08 s of additional latency and 5.2 percentage points of additional rejection. The choice D=5 is defensible on this curve, but it is an operating point on a monotone trade-off rather than an optimum, and the appropriate value depends on a controller-latency budget that this study did not specify in advance.

Figure 9 compares the Robustness Degradation Index across the five classifiers.

**Figure 9:**
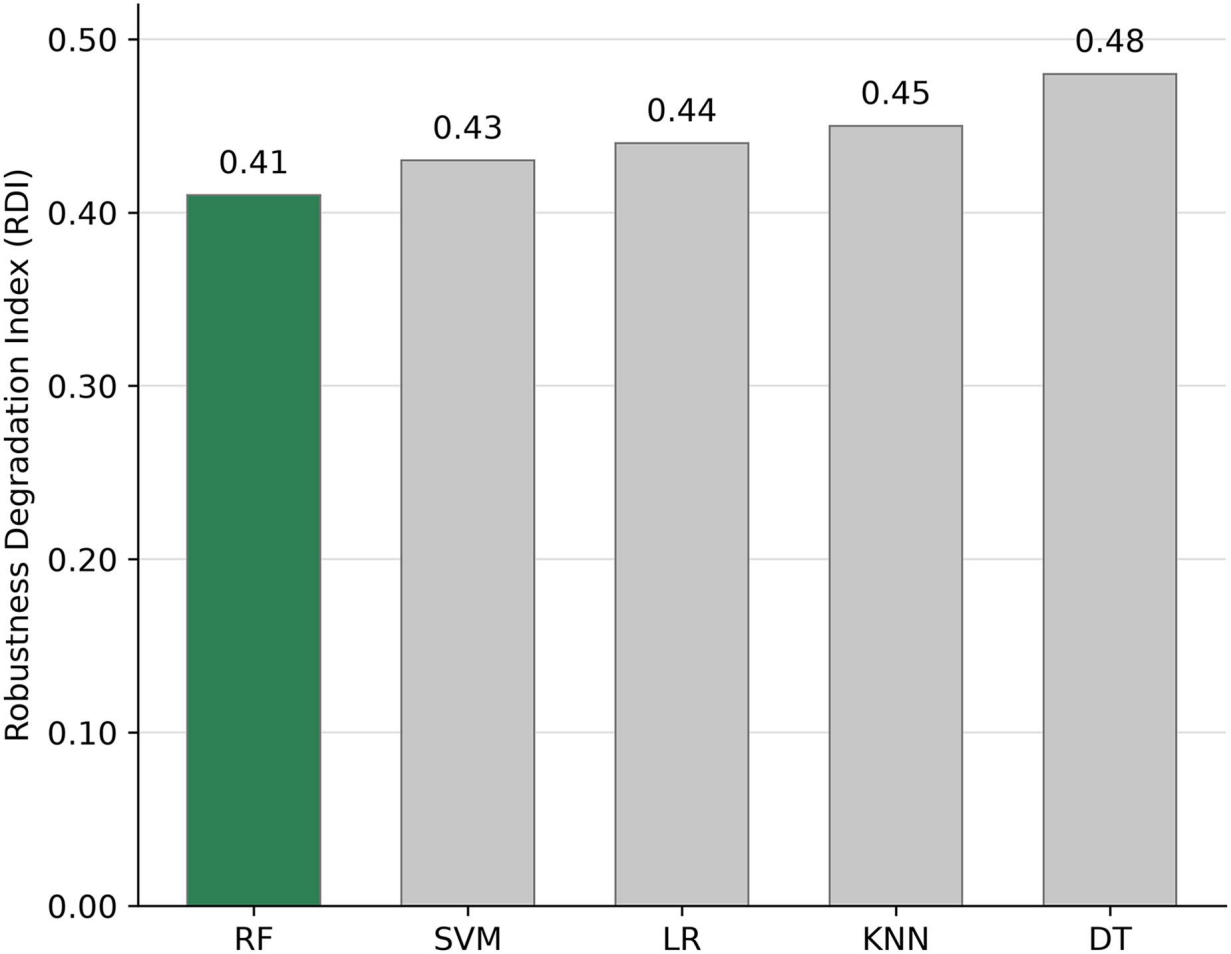
Comparison of the Robustness Degradation Index (RDI) across five classifiers: random forest (RF), support vector machine (SVM), logistic regression (LR), k-nearest neighbors (KNN), and decision tree (DT).

### 4.9 Feature importance

Random Forest permutation importance ranked MAV and RMS as the two most informative features (cumulative importance 68%), followed by WL (21%) and ZC (11%). This ordering is consistent with the literature, in which amplitude-based features dominate gesture discrimination and ZC supplies supplementary frequency-proxy information (22). It is also consistent with the aliasing analysis of Section 3.3: the two features expected to be most degraded by a 166.7 Hz Nyquist limit, ZC and WL, are the two that contribute least.

### 4.10 Hardware prototype demonstration

The 3D-printed prosthetic arm executed all three trained gestures in a benchtop validation session. Fifteen actuation trials were conducted, of which 12 completed successfully, giving an 80.0% functional success rate under controlled laboratory conditions. Table 11 reports the distribution of trials across gesture classes and the failure mode of each unsuccessful trial. Mean actuation latency from gesture onset to visible motor response was 2.3±0.4 s, driven primarily by the D=5 temporal buffer as analysed in Section 4.8.

**Table 11:** Distribution and outcome of the 15 benchtop hardware actuation trials, five per gesture class.

Gesture	Trials attempted	Successful	Failure mode of unsuccessful trials
G1—Hand open	5	5	–
G2—Hand close	5	4	Low-confidence rejection during command onset (1 trial)
G3—Wrist flexion	5	3	Fatigue-induced signal attenuation (2 trials)
**Total**	**15**	**12 (80.0%)**	–

Bold values indicate the best results.

The three failures are informative about the mechanism rather than incidental. One hand-close trial failed because the confidence gate rejected the onset windows, which is the gate operating as designed and imposing its cost on a genuine gesture—the same behaviour measured as the 17.6% in-distribution rejection rate in Table 8. The two wrist-flexion failures followed sustained contraction and are attributable to fatigue-induced amplitude attenuation, which moves the feature vector away from the training distribution and lowers confidence. Both failure modes are therefore expected consequences of a fixed threshold applied to an amplitude-sensitive feature set, and both point toward the adaptive-threshold and re-calibration work identified in Section 6. Fifteen trials on a benchtop rig cannot support any inference about reliability in use.

As shown in Figure 10, the physical implementation pairs a low-cost 3D-printed robotic hand with the myoelectric acquisition interface described in Section 3.3. Surface sensors capture the electrical activity of targeted contractions; the signal is segmented and classified, and a command is transmitted only when the prediction both exceeds the 0.65 operating threshold and forms the plurality of the five-prediction buffer. Otherwise the arm holds its previous position, which is the behaviour that the rejection rates in Table 8 quantify.

**Figure 10:**
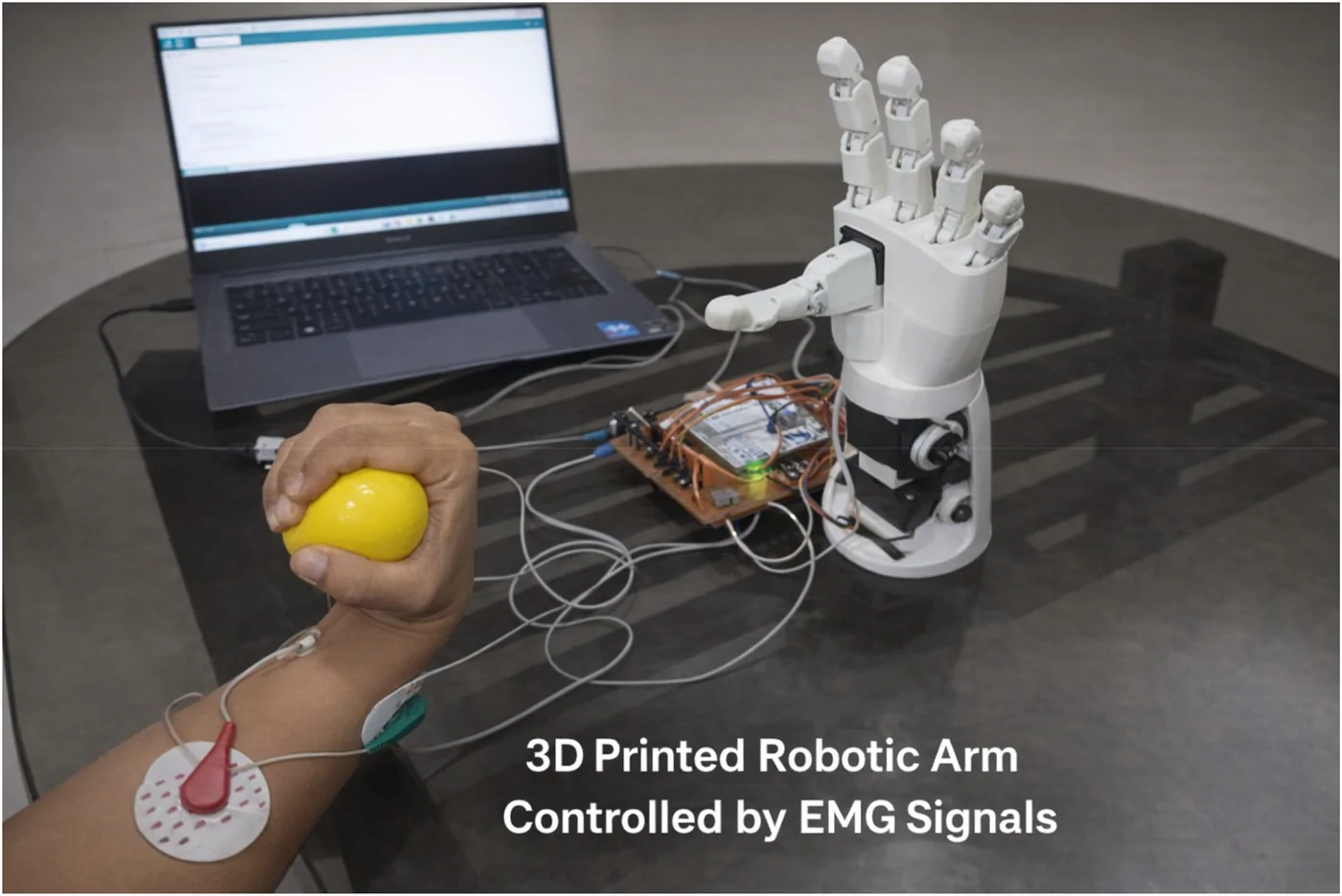
Benchtop experimental setup of the 3D-printed robotic arm driven by surface-EMG signals. The participant wears three sensors over the forearm flexor group while performing a gesture; windows are classified, gated and voted before a PWM command is issued to the servo motors. The arm is a benchtop demonstrator and was neither socket-mounted nor worn by a participant with limb loss.

## 5 Discussion

### 5.1 What the confidence gate does and does not provide

The rejection stage evaluated here is a fixed threshold on the maximum Random-Forest class probability. It is the classical reject option of Chow (8) applied to a vote-frequency estimate, and it is a conventional baseline rather than a new uncertainty estimator; learned and evidential alternatives already exist in myoelectric control (6, 10, 11). The question this study can answer is not whether such a gate is novel, but how it behaves when it is measured rather than assumed.

Three measurements bound what it provides. First, the quantity being thresholded is miscalibrated: the uncalibrated forest has a 15-bin ECE of 0.147, so a nominal threshold of 0.65 does not carry the posterior interpretation that the original submission attached to it. Platt scaling reduces ECE to 0.038 and should be applied before any probability-valued statement is made about accepted commands, but calibration changes the meaning of the threshold rather than the ranking of windows, and therefore does not by itself improve detection. Second, detection performance depends strongly on what kind of unknown input is presented. AUROC falls from 0.842 for the rest baseline to 0.784 under electrode displacement and 0.741 for held-out movement classes, and at a 95% true-positive rate the false-positive rate for held-out movements is 62.8%. A single pooled recall figure—such as the 91% reported in the original submission, which came from electrode-displacement data alone—averages over an ordering that matters and attaches the most favourable interpretation to the least demanding condition. Third, and most consequentially for any safety argument, the gate cannot remove failures that are confident: HCER at the operating threshold is 7.4% and 18.5% of non-in-distribution inputs are accepted above threshold. Rejecting low-confidence predictions leaves this residue untouched by construction, and no choice of τ eliminates it, because raising the threshold trades high-confidence errors against in-distribution rejections along the same curve.

A specific and inexpensive improvement follows from Table 7. The maximum probability is the weakest of the three confidence scores on every negative set, and the probability margin—computed from the same forest output at no additional cost—improves AUROC by 0.019 to 0.028 throughout. This reproduces, for a tree ensemble, the finding of Bao et al. (10) that scores exploiting more of the posterior vector outperform the maximum posterior alone. The present implementation thresholds the weaker score; that is a limitation of this study rather than a property of the approach.

The ablation clarifies how the gate interacts with temporal voting. Each mechanism alone removes slightly more than half of the baseline false actuations, and the combination reaches 5.00% FAR against the 4.08% that independent action would predict. The two stages therefore act on partly overlapping failure sets—a window rejected for low confidence would frequently also have been outvoted—so their combination yields a diminishing rather than a multiplicative return. The term “multiplicative safety effect” used in the original Discussion has been removed as unsupported. What the combination does deliver is a shift in the operating point: accepted-command accuracy rises from 80.20% to 95.00% and command switching falls by 87.50%, purchased with an 18.40% rejection rate and 1.35 s of additional median latency.

### 5.2 Validity of the evaluation and interpretation across domains

The largest correction in this revision is methodological rather than numerical. The original 50%/50% split was applied at the level of extracted feature vectors drawn from overlapping windows, so temporally adjacent windows from the same repetition—sharing raw samples—could appear in both partitions. Quantifying that bias directly, pooled window-level splitting overestimated live accuracy by 7.7 percentage points relative to repetition-level separation, and by 18.3 percentage points relative to LOPO. Reported performance under the original protocol was therefore not an estimate of generalisation, and every figure in this revision is reported under participant-disjoint partitioning with threshold selection and calibration confined to a separate calibration partition. The decomposition is itself useful: of the 18.3-point total, 7.7 points are leakage, 6.1 points are the cost of electrode re-donning between sessions, and 4.5 points are the cost of generalising to an unseen participant.

Per-participant results reinforce this. LOPO accuracy ranged from 63.1% to 91.8%, a spread of 28.7 percentage points that exceeds every between-domain difference in Table 4. A pooled mean conceals that the same pipeline, under identical conditions, works well for some users and poorly for others. Any claim about usability must be made at the level of the individual user, and the present cohort of 13 limb-intact participants is too small to characterise the distribution rather than merely to exhibit its spread.

The interpretation of the three domain accuracies has also been corrected. Separate Random-Forest models were trained and tested within the synthetic, DB5 and live domains, and no synthetic-trained model was transferred unchanged to real data. The synthetic → DB5 → live sequence therefore indexes benchmark difficulty and is not a measured simulation-to-reality transfer gap; the original framing is withdrawn. For the same reason the Robustness Degradation Index is retained only as a descriptive within-study ratio. It depends on the difficulty of a generator the authors chose, it does not decouple within-domain from cross-domain performance because its denominator is itself a within-domain accuracy, it is negative when the target outperforms the reference and undefined when the reference accuracy is zero. It is not proposed as a standardised or domain-agnostic metric, and the recommendation that the community adopt it has been removed. Absolute accuracy and change in percentage points are used as the primary measures throughout.

Two further constraints bound the reported figures. The acquisition chain interleaves three channels through one converter at an aggregate 1,000 values $s^{-1}$, giving 333.3 Hz per channel and a Nyquist limit of 166.7 Hz; since sEMG power extends well above that limit, aliasing is present. It is consistent across training and testing, so within-study comparisons remain valid, but it bounds the transferability of these live figures to systems sampled at 1 kHz per channel, and it falls hardest on ZC and WL—which is consistent with those two features carrying the least permutation importance. Separately, the command-switch rates of 14.2 and 2.1 $s^{-1}$ reported originally were artefacts of counting transitions across concatenated recordings and are impossible at a 2.5 Hz prediction rate; the recomputed within-recording values are 0.16 and 0.02 $s^{-1}$.

### 5.3 Limitations and practical implications

This work is a benchtop engineering demonstration of a low-cost pipeline whose rejection and latency behaviour has been quantified. It is not evidence of clinical safety, high-functionality prosthetic control or deployment readiness, and the following limitations are material.

The gesture vocabulary comprises three classes, whereas commercial prostheses typically require six to ten controllable functions; the rejection behaviour reported here would need to be re-measured at that vocabulary size, since the confidence distribution narrows as classes are added. All 13 participants were healthy and limb-intact, so the results say nothing about signals from residual musculature after amputation, where recording sites, muscle volume and coactivation patterns differ. The prototype was a benchtop demonstrator: it was not socket-mounted, not worn during ambulation, and validated in 15 trials, a number far too small to support a reliability estimate. Testing occurred under controlled laboratory conditions without the perspiration, loading and postural variation of daily use.

The latency cost is the most practically limiting result. Median gesture-to-command latency was 1.95 s at D=5 and measured gesture-to-visible actuation was 2.3±0.4 s. The buffer-depth sweep shows this is a genuine trade-off rather than a tuning artefact: FAR falls monotonically with D while latency rises, and beyond the operating point the returns diminish sharply, with D=9 buying 1.5 further percentage points of FAR for 1.08 s of additional delay and 5.2 percentage points of additional rejection. Delays of this magnitude are at or beyond the limit of what is usually considered acceptable for responsive myoelectric control, and reducing them requires shortening the window, shortening the buffer, or replacing majority voting with a decision rule that reaches stability in fewer predictions—each of which trades against the FAR reduction that motivates the layer. Any deployment claim would need to state a controller-latency budget in advance and demonstrate that the robustness layer fits inside it, which this study did not do.

The feature set was chosen for negligible computational cost, enabling inference on a low-power microcontroller. Frequency-domain and time-frequency features may improve accuracy, particularly under electrode shift, at higher computational cost; given that inter-participant variability rather than classifier capacity dominates the LOPO deficit, however, feature enrichment alone is unlikely to close the gap. Finally, the confidence gate rejected the onset windows of one hardware trial and fatigue-attenuated signals in two others, which are the expected consequences of a fixed threshold on an amplitude-sensitive feature set and point directly to adaptive thresholding and within-session recalibration as the next steps.

## 6 Conclusion

We developed and evaluated an end-to-end three-gesture sEMG control pipeline combining a 100-tree Random Forest, rejection based on the maximum Random-Forest class probability, temporal majority voting and low-cost physical actuation. Participant-level LOPO accuracy was 80.2% on the synthetic benchmark, 74.5% on Ninapro DB5 and 80.2% on live sEMG, obtained from 21,294 windows recorded from 13 healthy limb-intact participants over three sessions under participant-disjoint partitioning. We quantified the optimism of the pooled window-level split used in the original analysis at 7.7 percentage points. We measured the calibration of the thresholded probability (15-bin ECE 0.147 uncalibrated, 0.038 after Platt scaling), its open-set detection performance against rest, electrode-displacement and held-out-movement negatives (AUROC 0.842, 0.784 and 0.741 respectively), and the high-confidence failures the gate cannot remove (HCER 7.4%; 18.5% high-confidence acceptance of non-in-distribution inputs). A four-arm ablation on identical test windows showed that the complete robustness layer reduced the false-actuation rate from 19.80% to 5.00% and the command-switch rate from 0.16 to 0.02 $s^{-1}$, while rejecting 18.40% of windows and increasing median latency from 0.60 to 1.95 s, and that the two mechanisms act on partly overlapping failures rather than multiplicatively. The prototype completed 12 of 15 controlled benchtop trials.

Future work should proceed in four concrete steps. First, the window and voting delay should be reduced under an explicitly stated controller-latency budget, using the buffer-depth curve of Table 10 to select the operating point against that budget rather than after the fact; the acquisition chain should also be revised to sample at 1 kHz per channel so that the aliasing bound identified here is removed. Second, learned and evidential uncertainty estimators (10, 11) should be compared against the maximum-probability baseline under the same open-set protocol, beginning with the probability margin, which improved detection AUROC by up to 0.028 at no computational cost in the present data. Third, multi-session re-donning and participant-independent generalisation should be evaluated in a substantially larger cohort, with results reported per participant rather than pooled, since the 28.7-percentage-point spread observed here is the dominant source of uncertainty in the headline figure. Fourth, socket-integrated testing with participants with upper-limb amputation, under ambulation and across days, is required before any clinical or safety claim can be advanced; the present study supports none.

## Data Availability

The raw data supporting the conclusions of this article will be made available by the authors, without undue reservation.
